# Predictors of Bacterial Meningitis in Resource-Limited Contexts: An Angolan Case

**DOI:** 10.1371/journal.pone.0025706

**Published:** 2011-10-04

**Authors:** Cristina Lussiana, Sofia Vanda Lôa Clemente, Ivan Alejandro Pulido Tarquino, Isabel Paulo

**Affiliations:** 1 Infectious Diseases Laboratory, Hospital Divina Providencia, Luanda, Angola; 2 Hospital Divina Providencia, Luanda, Angola; University of Medicine & Dentistry of New Jersey - New Jersey Medical School, United States of America

## Abstract

**Background:**

Despite the great morbidity and mortality that childhood bacterial meningitis (BM) is experiencing in Africa, diagnosis of BM in resource-limited contexts is still a challenge. Several algorithms and clinical predictors have been proposed to help physicians in decision-making but a lot of these markers used variables that are calculable only in well-equipped laboratories. Predictors or algorithm based on parameters that can be easily performed in basic laboratories can help significantly in BM diagnosis, even in resource-limited settings, rural hospitals or health centers.

**Results:**

This retrospective study examined 145 cerebral-spinal fluid (CSF) specimens from children from 2 months to 14 years. CSF specimens were divided into two groups, according to the presence or not of a clinical diagnosis of BM. For each specimen, CSF aspect, CSF white blood cells (WBC) count, CSF glucose and protein concentration were analyzed and statistical analysis were performed. CSF WBC count ≥10/µl is no more a valuable predictor of BM. CSF protein concentration ≥50 mg/dl has a better sensitivity for BM diagnosis and when used with CSF glucose concentration ≤40 mg/dl, can help to diagnose correctly almost all the BM cases. An algorithm including CSF protein concentration, glucose concentration and WBC count has been proposed to rule out BM and to correctly diagnose it.

**Conclusions:**

In resource-limited health centers, the availability of a combination of easy-to-obtain parameters can significantly help physicians in BM diagnosis. The prompt identification of a BM case can be rapid treated or transferred to adequate structures and can modify the outcome in the patient.

## Introduction

Despite the introduction of a *Haemophilus influenzae* type b (Hib) vaccine, childhood bacterial meningitis (BM) remains one of the most important diseases responsible for infant mortality in developing countries [1]–[4]. In Africa annually more than 1 million patients acquire meningitis: 350000 die and at least 30% of survivors are left with sequelae [5]–[6]. The incidence of BM is lower in industrialized countries than in low-income countries, with a difference in incidence and mortality of 10 times [6]–[11]. The main etiological agent of BM in Africa in infants from 0 to 5 years has been Hib, followed by *Streptococcus pneumoniae* and *Neisseria meningitidis* [2]. Due to the difficult conditions in which African provincial hospitals are used to work, it is likely that incidence of BM is low-estimated and BM may be a major infant killer. As the lumbar puncture is not always performed in rural contexts, in Africa there could be an under-reporting of BM cases. We know that previous studies demonstrated that in Angola BM incidence was under-estimated: in 2008 a study performed in Luanda, Angolan capital, demonstrated how the mortality of BM patients in the last six years was no less than 50% [12]. This fact indicates how BM cases are under-reported and how BM diagnosis could be difficult in low-income countries.

In scientific literature several algorithms have been published: they use clinical and laboratory features to differentiate various forms of meningitis and to quickly diagnostic a BM. Various parameters have been investigated: haemoglobin, temperature, Glasgow coma score, Blantyre coma score, focal neurological signs, additional focus of infection, peripheral white blood cells (WBC) count, glycaemia, cerebral-spinal fluid (CSF) opening pressure, CSF WBC count, CSF polymorphs %, CSF protein concentration, CSF glucose, CSF-serum glucose ratio, positive bacterial culture and positive CSF latex agglutination. Unlucky none of these parameters has revealed an absolute clinical prediction of BM and the algorithms authors found have to been evaluated separately due to the context where studies were performed [13]–[15]. A positive bacterial culture from CSF specimens is of great use to unequivocally diagnose BM and to identify the etiological agent. In poor contexts or in rural health-centres a bacterial culture is not always easy to perform and low-facilities laboratories do not allow a correct diagnosis of BM. In poor settings and in the presence of low-equipped laboratories, easy-to-find parameters that can be highly predictive of BM could be more useful to diagnose BM.

In this study we aim to assess how basic laboratory parameters of CSF analysis can aid BM diagnostic even in the absence of a bacterial culture. The parameters we evaluated included CSF WBC count, CSF glucose concentration, CSF protein concentration and Gram staining.

## Materials and Methods

### Study setting

The study was conducted at Divina Providencia Hospital, Luanda (Angola), settled within the poorest suburbs of the capital. The hospital is a 110-bed municipal hospital; the emergency unit receives daily 200 patients. Patients may arrive from other health centres or other hospitals but most children arrive spontaneously from the great capital area.

### Study design

The study was a nested case-control study of patients who underwent a lumbar puncture in the emergency unit to diagnose or rule out BM between January 1, 2009 and December 31, 2010. Lumbar puncture was performed for patients presenting impaired consciousness, convulsions, prostration, meningismus or bulging fontanel. Exclusion criteria included age <2 months or >14 years, second lumbar puncture within 14 days, clotted samples, CSF red blood cell count >10000/µl and positive malaria thick smear. Medical records of patients admitted in the study were reviewed to extract additional information to ensure that diagnosis or exclusion of BM was correct and to verify that exclusion and inclusion criteria were met. Written informed consent was obtained from all patients or from their relatives if patient was unable to provide consent. Ethical approval for this study was obtained by the Institutional Review Board at our institution.

### Laboratory methods

CSF from the lumbar puncture at the time of admission was analysed. Processing of CSF specimens followed standard hospital procedures [16]. CSF analysis included aspect evaluation (divided into clear, low-cloudy and cloudy) using optical examination, WBC count per µl (determined manually with a modified Neubauer counting chamber), differential white cell count, glucose concentration and total protein concentration (evaluated using a standard spectrophotometric technique). Because of limitations in personnel, Gram staining was performed only if specimen was cloudy or if CSF WBC count was >10/µl or glucose concentration was <40 mg/dl [17]. Standard cut-off values were used as previously described in literature: CSF WBC count ≥10/µl, CSF glucose concentration ≤40 mg/dl and protein concentration ≥50 mg/dl [18]–[20].

### Statistical analysis

Data recorded manually were entered in duplicate into secure databases created in Microsoft Excel and Access and analysed using SPSS version 15. Confidence intervals (CI) were calculated using standard normal distribution; sensitivity, specificity, positive predictive value (PPV), negative predictive value (NPV) and odds ratio (OR) were calculated for each predictive variable [21].

## Results

A total of 179 CSF specimens were collected from January 1, 2009 and December 31, 2010. 34 CSF specimens were excluded: 32 were from patients >14 years, one had a red blood cell count >10000/µl and one patient was admitted for a second lumbar puncture within 14 days. The remaining 145 CSF specimens were divided into two groups: control group (n = 87) and BM group (n = 58). Diagnosis of BM was confirmed according to medical chart information. Patients' characteristics are represented in Table 1. Mean, standard deviation and 95% CI for each parameter evaluated in this study are represented in Table 2.

**Table 1:** Characteristics of patients of the 145 CSF specimens admitted in the study.

	Control group(n = 87)		BM group(n = 58)	
	n	%	n	%
**Age**				
O months to 12 months	8	(9.20)	3	(5.17)
13 months to 5 years	61	(70.11)	43	(74.14)
6 years to 14 years	18	(20.69)	12	(20.69)
**Sex**				
Male	47	(54.02)	26	(44.83)
Female	40	(45.98)	32	(55.17)
**Aspect**				
Clear	63	(72.41)	18	(31.03)
Low-cloudy	3	(3.45)	7	(12.07)
Cloudy	21	(24.14)	33	(56.90)
**WBC count**				
≥10/µl	28	(32.18)	46	(79.31)
<10/µl	59	(67.82)	12	(20.69)
**Glucose concentration**				
≤40 mg/dl	43	(49.43)	43	(74.14)
>40 mg/dl	44	(50.57)	15	(25.86)
**Protein concentration**				
≥50 mg/dl	32	(36.78)	51	(87.93)
<50 mg/dl	55	(63.22)	7	(12.07)
**Gram staining (n = 62)** *				
positive	2	(8.0)	25	(69.44)
negative	24	(92.0)	11	(30.56)

*For Gram staining n = 62. Gram staining was performed only for 26 specimens from control group and 36 specimens from BM group.

**Table 2:** Characteristics of evaluated parameters in control group and BM group.

a)	Control group(n = 87)			BM group(n = 58)		
	Mean	SD	95% CI	Mean	SD	95% CI
Age (years)	3.29	4.19	2.41–4.17	3.44	3.32	2.59–4.29
WBC count (/µl)	20.00	31.98	13.28–26.72	132.73	138.92	96.98–168.48
Glucose conc. (mg/dl)	56.29	32.37	49.51–63.07	31.15	22.37	25.39–36.90
Protein conc. (mg/dl)	53.56	42.08	44.72–62.41	165.21	147.63	127.21–203.20

b)	BM group versus control group	
	Difference of Means	Mean
WBC count (/µl)	112.73	3.44
Glucose conc. (mg/dl)	−25.14	132.73
Protein conc. (mg/dl)	111.64	31.15

*p<0.001.

a) Mean, Standard Deviation (SD) and 95% Confidence Intervals (CI) of evaluated parameters. b) Difference of Means and 95% Confidence Intervals of evaluated parameters.

All the children who underwent a lumbar puncture at Divina Providencia Hospital belonged to a low socioeconomic group, which represents 90% of Luanda population [22]. Malnourishment was common, at 35%, being severe in 15% of cases and moderate in 85% of cases. Hence hygiene conditions were also very poor with low or null access to drinkable water and no adequate sewage disposal. CSF WBC count mean in control group is 20.00/µl (95% CI is from 13.28 to 26.72) whilst CSF WBC count mean in BM group is 132.73/µl (95% CI is from 96.98 to 138.48). CSF glucose concentration mean in control group is 56.29 mg/dl (95% CI is from 49.51 to 63.07) whilst CSF glucose concentration mean in BM group is 31.15 (95% CI is from 25.39 to 36.90). CSF protein concentration mean in control group is 53.56 mg/dl (95% CI is from 44.72 to 62.41) whilst CSF protein concentration mean in BM group is 165.21 (95% CI is from 127.21 to 203.20).

**Figure 1:**
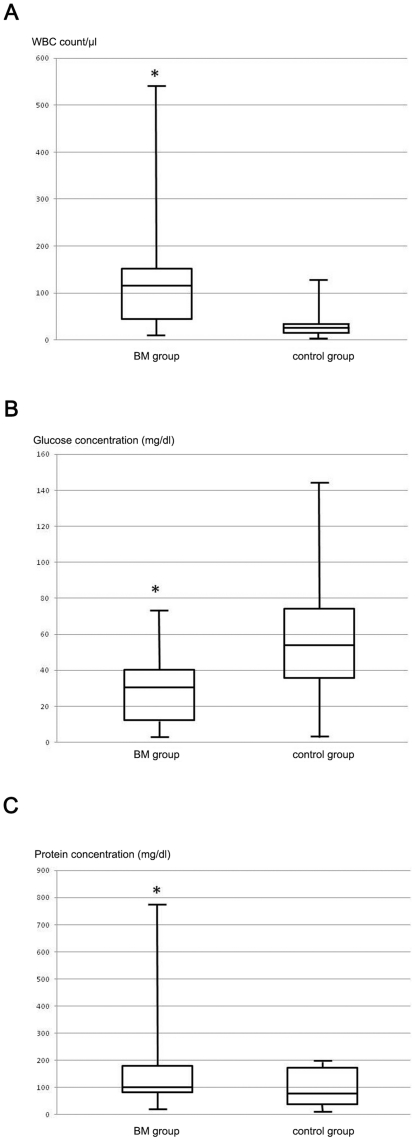
Graphical representations of minimum, maximum, mean, upper and lower quartile of examined parameters in control and BM group. a) WBC count, b) glucose concentration, c) protein concentration. For each parameter the difference of means is statistically significant. * = p<0.001.

There were no significant differences in mean age between control group and BM group and the distribution of males and females into the two groups is almost equal. Case fatality ratio among children diagnosed with BM was 15.52% (n = 9, 8 males and 1 female). When compared, the difference in CSF WBC count mean between control and BM group is statistically significant (difference in WBC count means is 112.73 WBC/µl, 95% CI is from 76.35 to 149.11, p<0.001); also the difference in CSF glucose and protein concentration means are statistically significant (difference in CSF glucose concentration means is −25.14 mg/dl, 95% CI is from −34.04 to −16.24, p<0.001; difference in CSF protein concentration means is 111.64 mg/dl, 95% CI is from 72.63 to 150.65, p<0.001). For each parameter we examined, Figure 1 shows graphically the distribution of the values within the two groups. Minimum, maximum, mean, upper and lower quartiles are here represented; each parameter reveals a statistically significant difference between the mean in control group and BM group (p<0.001).

Using standard cut-off values [18]–[20], we also calculated the OR for each parameter (Table 3). The OR of CSF WBC count ≥10/µl for BM is 8.08 (95% CI is from 1.36 to 47.81, p = 0.01); the OR of CSF glucose concentration ≤40 mg/dl for BM is 2.93 (95% CI is from 1.17 to 7.33, p = 0.102; the OR of CSF protein concentration ≥50 mg/dl is 12.52 (95% CI is from 1.46 to 107.66, p<0.01).

**Table 3:** Diagnostic characteristics for predicting BM.

	OR (95% CI)	Sensitivity, %	Specificity, %	Positive Predictive Value, %	Negative Predictive Value, %
All the parameters	9.79 (1.40–78.23)	58.62	87.36	75.56	76.00
CSF WBC count ≥10/µl	8.08 (1.36–47.81)	69.31	67.82	62.16	83.10
CSF glucose conc. ≤40 mg/dl	2.93 (1.17–7.33)*	74.14	50.57	50.00	74.58
CSF protein conc. ≥50 mg/dl	12.52 (1.46–107.66)	87.93	63.22	61.45	88.71
CSF WBC count ≥10/µl and CSF glucose conc. ≤40 mg/dl	5.83 (1.30–26.17)	60.34	79.31	66.04	75.00
CSF WBC count ≥10/µl and CSF protein conc. ≥50 mg/dl	11.59 (1.44–93.25)*	68.97	83.91	74.07	80.22
CSF glucose conc. ≤40 mg/dl and CSF protein conc. ≥50 mg/dl	16.41 (1.52–177.52)*	72.41	86.21	77.78	82.42

*p<0.01.

Using a combined analysis we calculated the OR of a combination of two parameters: the OR of CSF WBC count ≥10/µl and glucose concentration ≤40 mg/dl is 5.83 (95% CI is from 1.30 to 26.17, p<0.05); the OR of CSF WBC count ≥10/µl and protein concentration ≥50 mg/dl is 11.59 (95% CI is from 1.44 to 93.25, p<0.01); the OR of CSF glucose concentration ≤40 mg/dl and protein concentration ≥50 mg/dl is 16.41 (95% CI is from 1.52 to 177.52, p<0.01).

The OR of all the three parameters for BM is 9.79 (95% CI is from 1.40 to 68.23, p<0.01).

We also calculated sensitivity, specificity, PPV and NPV for these parameters and their combinations (Table 3). The NPV describes the post-test probability that the child does not have BM and is a valuable indicator of how well the test rules out BM. The PPV is used to indicate the probability that the patient really has BM in case of a positive test. These two association measures can aid to choose the best predictors of BM when compared among them.

Gram staining results were analyzed separately. Gram staining was only performed for 62 CSF specimens due to limitations in personnel. Gram-staining revealed a different percentage of Gram positive and Gram negative bacteria. In total 27 specimens resulted positive for Gram staining: 70.37% were identified as diplococcos Gram-positive while 26.63% resulted in coccos Gram-negative. Using a clinical diagnostic of BM as gold-standard, the sensitivity and specificity of Gram staining were respectively 69.44% and 92.31%; PPV and NPV were respectively 92.59% and 68.57%.

## Discussion

Physicians are likely to suspect a BM in children when CSF WBC count is far from normal values. When no other clinical or biochemical factors consistent with BM are identified, these children are often hospitalized and treated empirically with antibiotics [23]. As previously described in literature [24] BM can occur with mild or no CSF pleocytosis. Our study supports this hypothesis and demonstrates that a WBC count >10/µl is not a strong predictor of BM as it has an OR of 8.08 and a low PPV (62.16%). This can also be due to the fact that WBC count mean in control group is higher than 10/µl so the cut-off value of 10/µl could not be a good cut-off point. But even when we set the WBC count cut-off at 30/µl, the OR is 7.87 (95% CI is from 1.36 to 45.55, p<0.01) and the PPV is 68.42%: this is higher than the PPV of a WBC count cut-off of 10/µl, but the PPV is still poor. In our study 20.68% of BM cases occurred in CSF samples with a WBC count lower than 10/µl: all of these children had at least one factor that independently indicates that the child was at risk of BM (high CSF protein concentration or low CSF glucose concentration). Due to the fact that CSF WBC count ≥10/µl or ≥30/µl does not demonstrate a good sensitivity and due to the high ratio of children that underwent to BM with a WBC count lower than 10/µl, we cannot use CSF WBC count to confirm or rule out BM. We have to consider other possible markers of BM.

CSF protein concentration has a better OR (12.52) than CSF WBC count has. It also has the highest NPV within the examined parameters (88.71%). These results can demonstrate that CSF protein concentration lower than 50 mg/dl is likely to exclude a BM. Even with a sensitivity of 87.93%, the PPV of CSF protein concentration alone is poor (61.45%), so we discourage the use of CSF protein concentration as single marker to predict BM.

When the parameters are examined together as predictors of BM, the best OR belongs to CSF glucose and protein concentration (16.41). This couple has also the best PPV of all the combinations we examined and it seems to be a good marker of BM in our cases. The sensitivity and the specificity of this couple are respectively 72.21% and 86.41%: when used together these two BM markers can aid to correctly identify BM in 72.21 of 100 cases. Its NPV (82.42) also demonstrates that in resource-limited settings the evaluation of only two parameters can aid significantly to rule out BM. When other BM-associated factors are also present, the co-presence of high protein concentration and low glucose concentration is a strong indicator of BM.

In this study CSF protein concentration has the best sensitivity (87.93%), so we can start from here to construct an algorithm to be used in resource-limited contexts. The presence of CSF protein concentration higher than 50 mg/dl indicates a high probability of BM. Notwithstanding, 12.07% of BM cases occurred in CSF samples with protein concentration lower than 50 mg/dl. 86% of these cases had CSF WBC count higher than 10/µl whilst 50% of them had CSF glucose concentration lower than 40 mg/dl. CSF glucose concentration has a better sensitivity than CSF WBC count so we can add CSF glucose concentration to our algorithm to aid to identify possible CSF specimens with BM.

In resource-limited settings we advice to use CSF protein concentration as initial predictor of BM, followed by CSF WBC count and CSF glucose concentration. This is in fact the sorting order of parameters that demonstrate the better OR to diagnose BM. If CSF protein concentration is lower than 50 mg/dl, we suggest examining CSF WBC count: if it is higher than 10/µl and/or if CSF glucose concentration is lower than 40 mg/dl, there is a strong suspect of BM. To evaluate the efficacy of the algorithm we proposed, we calculated its specificity and sensitivity for the 145 CSF specimens collected. The application of our algorithm in diagnosing BM resulted in the identification of 57 out of 58 CSF specimens of BM group. This means that our algorithm has a 98.28% sensibility. Our algorithm revealed also 96.55% specificity because it identified 3 out of 87 CSF specimens positive to BM diagnosis although they belong to the control group. These low percentages of false-negative and false-positive results demonstrate the fine accuracy our algorithm has when it is used to support the BM clinical diagnosis.

We have also evaluated the possibility to include other parameters in our algorithm. The only marker that is easy-to-perform in resource-limited settings is CSF aspect. Due to the fact that CSF aspect evaluation is prone to inter-observer variability, we did not include this parameter in our algorithm. We are aware that other more accurate parameters as CSF-serum glucose ratio, positive bacterial culture and positive CSF latex agglutination could notably improve the accuracy of our algorithm, but these markers required a well-equipped laboratory and, consequently, are not applicable to rural African health centres.

The limit of our study is also represented by the fact that CSF glucose and protein concentration can be altered by other factors other than BM. The most accurate way physicians have to exclude a BM is a negative bacterial culture; due to the fact that resource-limited settings do not possess facilities to perform a bacterial culture, our algorithm can not reach 100% specificity if compared to bacterial culture. CSF glucose level is lower than normal in BM, meningealcarcinoma and sometimes in intracranial haemorrhage. CSF protein level can increase in BM, brain tumors, diabetes, multiple sclerosis, syphilis and guillian-Barre syndrome. In African contexts little is known about epidemiology of these diseases and even littler is known about the correlation between these diseases and CSF parameters. For that reason we strongly suggest that BM diagnosis has always to be accompanied by clinical evaluation in order to exclude diseases that can be responsible for altered CSF parameters and to assess the reliability of BM.

When a Gram staining is available it can help physicians optimize their decision making with respect to possible hospitalization and antibiotic treatment. In our study only 62 CSF specimens were analyzed for Gram staining. Gram staining resulted in a good specificity (92.31%) and a poor sensitivity (69.44%), reflected also by a low NPV (68.75%) and a good PPV (92.59%). These values reflect the fact that Gram staining could aid to diagnose BM, but the use of Gram staining alone can lead to the lost of positive BM specimens due to the high proportion of false-negative results. Our positive Gram staining specimens revealed a higher percentage of Gram-positive bacteria compared to Gram-negative bacteria. As it was not possible to perform a bacterial culture, we can only speculate on the identity of possible pathogens responsible for BM. *Streptococcus pneumoniae* –a Gram-positive bacteria- is likely the most important pathogen responsible for BM in our sample population as it appeared in 70.37% within positive Gram-staining specimens; *Neisseria meningitidis* –a Gram-negative bacteria- is less present (26.63%). These findings are consistent with other results reported in Angola after the introduction of the Hib vaccine [12], [22]. As interpretation of Gram staining requires trained biologists or lab-technicians we discourage to use routinely Gram staining in BM diagnosis and we suggest that interpretation of Gram staining results has to be accompanied by other BM predictors as CSF WBC count, CSF glucose or protein concentration.

In resource-limited settings, the availability of CSF parameters that can be calculated even in basic laboratories can aid physicians to diagnose or rule out BM. As previously suggested [24], CSF WBC count higher than 10/µl or 30/µl is no more a valid predictor of BM and it has to be substituted by other more reliable markers, above all in area where meningitis has a high morbidity and mortality.

Accurate diagnosis and prompt treatment in African hospitals remain a challenge. We attempted to produce a simple diagnostic rule using basic laboratory tests, available in most health care facilities in Angola. Because it was retrospective in nature and because of the low number of CSF specimens we analyzed, our study has certain limitations that can be overwhelmed only if our findings and our algorithm are accompanied by clinical features. Our findings regard some easily measurable variables and they can be of value only in resource-limited countries. When interpretation of Gram staining results is doubtful and when bacterial culture is no available, we suggest using the easy algorithm we provide in order to diagnose correctly BM. Meningitis caused by other pathogens, as fungi or viruses, present chemical values that differ from the ones here described. Viral meningitis use to have a normal value of CSF glucose concentration [25] so the algorithm we proposed is only applicable to BM.

We insert our study in a more comprehensive effort that other scientists are doing in Africa with the aim to improve quality and rapidity of BM diagnosis. Above the use of a valid diagnostic test and a predictive combination of CSF characteristics, encouraging parents to seek medical cares for their children promptly, transferring patients without delay and ruling out cerebral malaria are some of the tools at hand today to improve the prognosis of childhood bacterial meningitis in sub-Saharan Africa.
